# Illness conversations: Self-disclosure among children and youth with chronic illnesses

**DOI:** 10.1177/17423953221110152

**Published:** 2022-07-05

**Authors:** Tharanni Pathmalingam, Fiona J Moola, Roberta L Woodgate

**Affiliations:** 1 School of Early Childhood Studies, 7984 Toronto Metropolitan University, Toronto, Canada; 2 School of Early Childhood Studies, Faculty of Community Services, 7984 Toronto Metropolitan University, Toronto, Canada; 3 Holland Bloorview Kids Rehabilitation Hospital, 7984 Ryerson University, Toronto, Canada; 4 Dalla Lana School of Public Health, University of Toronto, Toronto, Canada; 5 Rehabilitation Sciences Institute, University of Toronto, Toronto, Canada; 6 Rady Faculty of Health Sciences, College of Nursing, 8664 University of Manitoba, Winnipeg, Canada

**Keywords:** children, youth, disclosure, chronic illness

## Abstract

**Objective:**

Illness disclosure refers to when individuals with chronic illnesses make decisions to tell others about their disease. There is a lack of research on the complexity of disclosure among children and youth with chronic illnesses. We conducted a review of the literature to understand the process of disclosure among children and youth with chronic illnesses in the context of peer-based relationships.

**Methods:**

A narrative review was completed using three databases. The search yielded 11 articles that utilized various research designs.

**Results:**

Most of the literature was qualitative in nature. Most children and youth engaged in non-disclosure and concealment which is born out of fears of discrimination. Fewer children and youth engaged in unplanned disclosure, passive disclosure, partial disclosure, and open disclosure. Children and youth carefully consider who they are disclosing to and perceptions about other peoples’ responses strongly impact disclosure. Children and youth disclose verbally, behaviorally, and in written form. Disclosure is associated with both positive and negative effects, such as confidence, self-advocacy, and distress.

**Discussion:**

Health providers and researchers should critically support disclosure and empower children and youth with the resources they need to be agents in their own disclosure decisions.

## Introduction

In moral philosophy, the values of honesty and transparency are valued in many cultures.^1^ With regards to disclosure and childhood chronic illness in North American and European countries, most of the literature has focused on when parents or physicians should disclose the condition to the child. Before 1948, chronically ill children were not given any information about their ailments due to a discourse of protectionism and the notion that any illness information could harm them.^2^ With the growing child rights movement of the 1980s and the recognition that non-disclosure may be harmful to children, the disclosure discourse began to change. Best practice today recommends telling children about their illness in developmentally appropriate ways.^2^

Unfortunately, the disclosure literature offers little guidance on the nature and process of chronically ill children and youth’s disclosure to peers. Thus, the disclosure literature is adultist, maintaining a primary focus on parents’ decisions. In this narrative review, we address these glaring lacunae in the disclosure literature by exploring how children and youth with chronic illnesses disclose their conditions to peers. The purpose of this narrative review is to investigate how children and youth with a chronic illness make decisions to disclose their diagnosis to their peers. Here, peers are understood to be individuals of similar age and/or developmental stages who share common spaces.^3^

Childhood chronic illnesses can be considered as any illness that is incurable or lasts a duration of three months or longer, involves greater health services that extend beyond routine care, and limits functioning.^4^ Despite increased survival, the rarity of childhood illness, and reduced mortality over time, these children may experience great hardship and psychosocial morbidity.^5^ For instance, children and youth with chronic illnesses report experiencing social isolation, bullying, and reduced self-efficacy.^5–7^ These impacts are crucial to investigate since school-age children and adolescents are at a time in their lives when they begin to seek social acceptance and opportunities from systems outside of their family.^8^ However, not all outcomes are adverse and positive outcomes such as resiliency and growth have also been observed.^9^ Both positive and negative outcomes can have short-term and long-term impacts on children’s psychosocial well-being.

One process that heavily impacts the psychosocial wellbeing of children and youth with chronic illnesses is self-disclosure. Self-disclosure is a complex, dynamic, and continuous process in which individuals carefully select who, what, and how they tell others about their illness.^10^ Factors such as time, setting, anticipated reactions, previous experiences, and the strength of friendship all play influential roles in whether and how children disclose their illness.^11–13^ Some of the reasons why youth choose to disclose their illness are associated with wanting understanding, acceptance, and support from those who have similar experiences.^11^ On the other hand, the decision to withhold illness information includes fear of rejection and stigmatization.^6,11–13^ The literature illustrates a favor towards self-disclosure because it can result in psychosocial benefits which consist of sharing new identities, strengthening relationships, and maintaining self-esteem.^11–13^ Despite the importance of the existing literature, there is a lack of evidence on how children and youth with chronic illnesses engage in this complex process. Indeed, studying disclosure among children and youth is of critical importance given that disclosure is known to impact important interpersonal and intrapersonal processes, such as perceptions of acceptance, and stigmatization.^4,14^ The purpose of this narrative review is to understand how children and youth with chronic illnesses disclose their illness in the context of peer-based relationships.

## Methods

### Rationale for a narrative review

It is important to conduct a narrative review of literature given the propensity of narrative reviews to capture rich, story-like information on the intricacies and nuances of peer-based relationships.^15,16^ Indeed, the potential for rich narrative and storying of the literature may be lost by reliance on more formal review types, such as scoping reviews or systemic reviews. The narrative-review type has the potential to capture the subtleties, nuances, and complexity of disclosures in the context of peer-based relationships. Additionally, this review type is incredibly useful when trying to synthesize diverse disciplines and methodological traditions.^16^

### Search strategy

The search strategy was developed and guided by workshops, research team discussions, meetings with a librarian, and reading other narrative reviews. These practices resulted in the use of three databases which included MEDLINE, PsycINFO, and EMBASE. These databases were found on Toronto Metropolitan University’s Library and Archives webpage and were chosen to encompass the clinical and psychological viewpoints of the research purpose. Initially, we aimed to investigate the disclosure practices that occur among children with cancer. Due to the extremely limited results, we had to re-evaluate our research purpose and expand our search to include all chronic illnesses and the youth population. A set of keywords were used for our search including, disclosure, chronic illness, child, adolescent, youth, peer, and friend. The keywords were chosen to gather articles that capture most if not all aspects of the research purpose. Cancer was also a keyword. Although we revised our original purpose, we still wanted to search for cancer-specific articles, as they are of particular interest. The keywords were combined with the conjunctions ‘and’ or ‘or’. The database code of truncation (*) was also used to gather relevant articles in a single search. For example, one of our searches was disclos* AND (chronic illness* or chronic disease* or chronic condition*) AND (peer* or friend*). Additionally, filters were applied to narrow the search criteria. These filters consisted of articles written in English and specified age categories. These categories slightly differed in each database. Generally, we used the age category filters of child, adolescent, and young adult, which resulted in an age search range of 0–29.

### Screening process

The screening process was completed by the first author. The author documented the searches that were used and articles of interest. These documents were shared in regular meetings with the second author where we discussed major decisions and any questions we had regarding the process of developing and writing this review. The inclusion criteria was developed prior to conducting the search and readjusted along the way. This criteria consisted of articles that: (1) were peer-reviewed and full-text; (2) written in the English language (3) were published after 2000; (4) had participants with chronic illness; (5) had participants under the age of 25; and (6) were focused on disclosure among peers. Given that childhood chronic illnesses are rare and recruitment into studies is extremely challenging, the application of age-specified filters, and our desire to stay close to the child population, we wanted to maintain an inclusive definition of children and youth, up until the age of 25 years.^17^ The existing literature consists of many articles that provide recommendations on when and how parents should provide children with developmentally appropriate information about their chronic illness.^18^ Children as young as three years old may be able to understand their illness although they may regard death as reversible. However, we found a lack of empirical guidance on when children are able to disclose their condition to peers. Indeed, the lack of developmentally appropriate disclosure guidance for young children may speak to the underdeveloped disclosure literature.

We operationalized the concept of disclosure as a process in which individuals carefully navigate who, what, where and how they tell others about their illness. This study was inclusive of various research approaches including, qualitative, quantitative, mixed-methods and reviews of literature. All other publications that did not fit this criterion were excluded. We undertook a thematic analysis of the final pool of selected articles following the steps outlined in previous scholarship.^16,19^ First, the main findings from each study in the 11 articles were extracted into a table. These findings were coded using a numeric indicator. Then, common numeric indicators were grouped together into larger themes that were common across all of the studies. After the seven themes were generated, we ensured that each theme was conceptually distinct from other themes so that data in that category represented a novel or unique theme. We verified the themes based on developing consensus with the research team.

### Selecting articles

**Figure 1.**
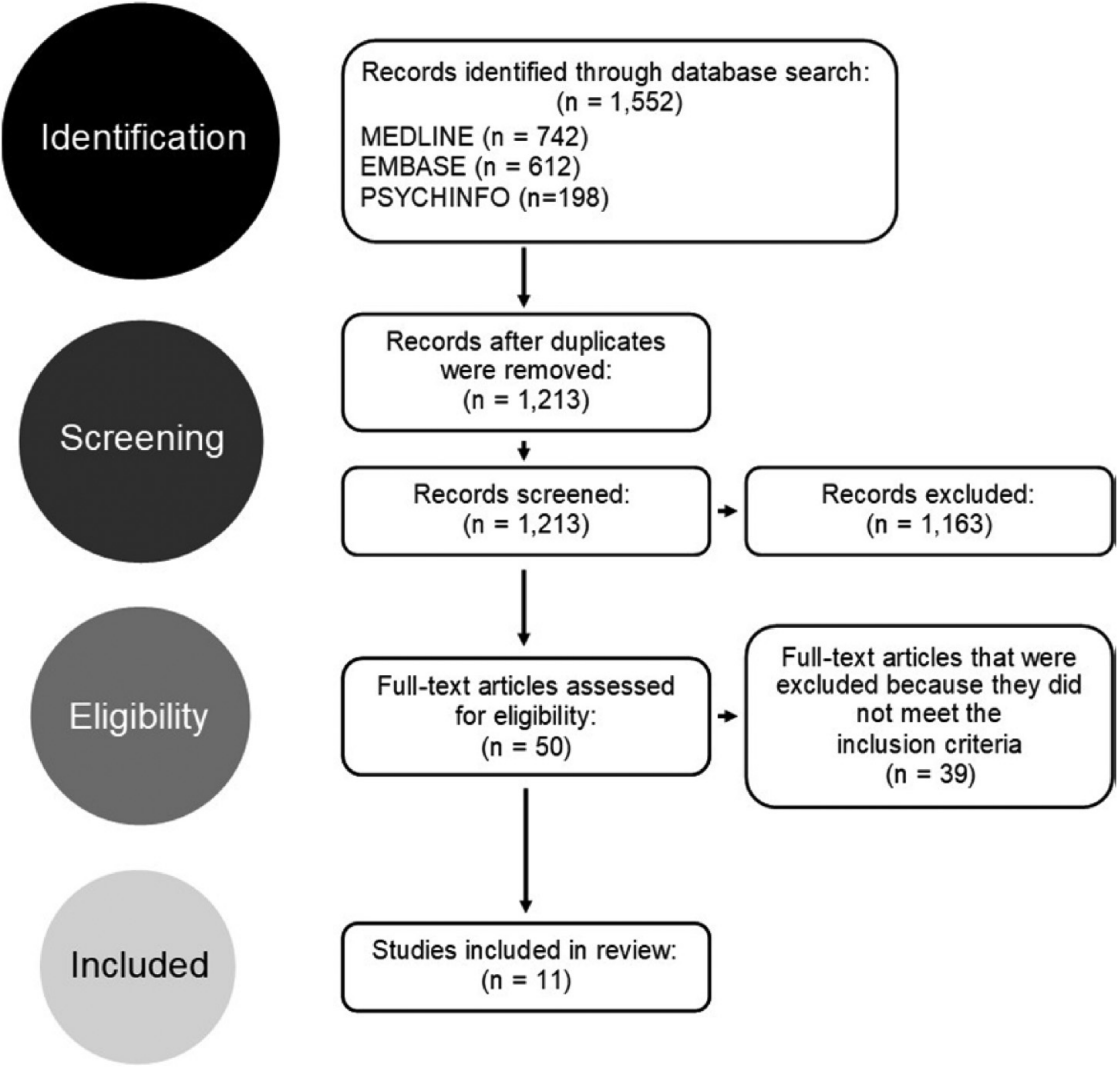
PRISMA flow diagram describing the selection procedure for this narrative review.

A total of 1552 articles were found regarding disclosure practices among peers (Figure 1). This number decreased to 1213 after removing duplicate articles using the Mendeley reference manager software. To determine whether the remaining articles were relevant to the study, both the title and the abstracts were read. Those that were not related to the research purpose were removed. All, but 50 articles were excluded from the search. For the remaining articles, the full article was read to determine whether the articles met the inclusion criteria and provided sufficient information relating to our research purpose.

## Results

A total of 11 articles were identified to be eligible for this study (Table 1). These articles ranged from the year 2009 to 2020. The articles all aimed to identify how and why children, adolescents, and young adults disclosed their chronic illness to peers.

**Table 1.** Review of articles investigating the disclosure process among children and youth.

Title, Author(s) Year of Publication	Chronic Illness Type	Purpose	Country	Participants	Methods/Methodology	Key Findings
To tell or not to tell: A qualitative interview study on disclosure decisions among children with inflammatory bowel disease Barned, Stinzi, Mack, *et al*. (2016)^13^	Inflammatory Bowel Disease (IBD)	To gain insight as to how Canadian children and adolescents decide if and how they tell their illness to others	Canada	25 participants between the ages of 10 and 17 years old, who have IBD 13 = male 12 = female	A qualitative study involving Interviews	- Concealment was the default form of disclosure for many participants - Some chose to share a few aspects of their illness - Open disclosure occurred with peers who participants knew for a long time or trust - The extent of knowledge a participant had about their illness was related to when they told others about their illness - Predictions about reactions, such as judgement and teasing, influenced whether children disclosed their illness - How the disease was linked to their identity was also concerning for participants - A few participants struggled with navigating conversations regarding their illness
To tell or not to tell: A systematic review of the disclosure practices of children living with epilepsy and their parents Benson, O'Toole, Lambert, *et al*. (2015)^20^	Epilepsy	To examine disclosure practices, enablers and barriers to disclosure, outcomes of disclosure, and the connection between disclosure and other variables	Review conducted in Ireland	Only data related to children’s experience of the disclosure process was used for this review Self or by proxy reported responses from 1429 children with epilepsy between the ages of 0 and 18 and 1838 parents of children with epilepsy	A systematic review containing qualitative, quantitative and mixed- methods designs	- Concealment or non-disclosure was related to social exclusion, teasing, bullying, negative perceptions of epilepsy from self and others, previous experience of negative reactions, and fear of being treated differently - Voluntary disclosure, selective disclosure, and unplanned revelations occurred among youth - Reasons for open disclosure included a desire for help and support from others - Youth reported experiencing positive reactions from peers after disclosure such as understanding, support, and taking an interest in learning about the illness - Open disclosure resulted in increased levels of advocacy and acceptance, whereas concealment resulted in embarrassment and misunderstanding
“I Don’t Like to Make a Big Thing out of It”: A Qualitative Interview-Based Study Exploring Factors Affecting Whether Young People Tell or Do Not Tell Their Friends about Their IBD Carter, Rouncefield-Swales, Bray, *et al*. (2020)^10^	Inflammatory Bowel Disease (IBD)	To contribute knowledge in the area of disclosure by exploring the impact IBD has on social relationships and psychological functioning	United Kingdom	31 participants ranging in the ages of 14 and 25 who have IBD 16 = male 15 = female	A qualitative study which utilized interviews, friendship maps, and photographs	- Reasons for non-disclosure was related to wanting privacy and avoiding embarrassment regarding disease - A few participants engaged in preventive disclosure - The decision to disclose an illness was related to wanting support, warning others, and wanting to educate others - Participants reported disclosing their illness to peers who they described as close and trusting - Content of what participants disclosed was dependent on who they disclosed to, the purpose of disclosure and confidence regarding illness - Considerations such as settings and verbal or written disclosure were reported by a few participants - Mixed reactions to disclosure were stated by participants
Disclosure and sickle cell disorder: A mixed methods study of the young person with sickle cell at school Dyson, Atkin, Culley, *et al*. (2010)^21^	Sickle Cell Disorder	To investigate the extent to which students disclosed their conditions at school and to explore the assumption that disclosure at school improves school experiences.	England	Individuals aged 4 to 25 Questionnaire: 281 = male 288 = female Interview: 19 = male 21 = female Most participants were of Black African/Caribbean descent	A mix-method study involving 569 questionnaires and 40 taped interviews	- Non-disclosure was related to negative experiences including social isolation and teasing - Disclosure was associated with support, empathy, and help from school peers
Stigma and Disclosure in Patients with Inflammatory Bowel Disease Guo, Rohde, & Farraye (2020)^14^	Inflammatory Bowel Disease (IBD) & other chronic illnesses	To inform others about the public perceptions and stigmas that are associated with IBD and their impact on patients	Review conducted in United States	Only information involving children, adolescents, and young adults was used for this review	A Narrative Review	- Patients reports avoiding embarrassment and the fear of being viewed differently as reasons to not disclose their illness to peers - Disclosure typically occurred with those who were described as emotionally close or expressed an interest in the illness - A few participants reported that disclosure occurred out of necessity or when peers asked questioned their behavior - Those who had their illness involuntarily disclosed risk suffering from negative stigma and embarrassment - Selective disclosure and protective/preventive disclosure was used to navigate and control negative reactions regarding their illness
Why do children and adolescents with epilepsy disclose or not disclose their condition to their friends? Jeschke1, Woltermann, Neininger, *et al*. (2020)^22^	Epilepsy	To explore why children and adolescents with epilepsy disclosed their condition to their peers	Germany	101 participants ranging in the ages of 6 to 18 years old 57 = male 44 = female	A qualitative study which utilized interviews	- The majority of the participants reported telling their friends about their illness - The participants who reported to not disclosing their illness illustrated mixed responses when asked about the outcomes that can occur after disclosure - Trust, honesty, support during emergency situations, and questions regarding behavior were a few of the reasons why children chose to disclose their illness - Reactions to disclosure included astonishment, empathy, and request for more information
Living a secret: Disclosure among adolescents and young adults with chronic illnesses Kaushansky, Cox, Dodson, *et al*. (2017)^6^	Chronic Illnesses: spina bifida, rheumatology, cardiology, cystic fibrosis, and renal transplant/dialysis.	To examine how and why individuals with chronic illnesses, both visible and invisible, shared their diagnosis with others	United States	25 adolescents and young adults aged 18 to 21 participated in this study 11 = male 14 = female 80% of participants were from Latino backgrounds	A qualitative approach involving interviews	- Generally, participants reported not disclosing their illness to peers unless it was a necessity - In some peer groups, participants chose to share with others who had chronic illnesses, as they perceived these peers to be more understanding of their experiences - The decision to disclose an illness was dependent on the visibility of the illness, the perceived reactions from peers, and practical needs
Stigmatising feelings and disclosure apprehension among children with epilepsy Lambert, Gallagher, O'Toole, *et al*. (2014)^23^	Epilepsy	To bring awareness to the impact stigma regarding epilepsy has on children communicating their illness to their social environments	Review conducted in Ireland	Only information about children was used for this review	Narrative review	- Identified and defined 5 types of disclosure; concealment, unplanned revelation, selective/partial disclosure, preventive disclosure, and open disclosure - Studies state that children experience apprehension, anxiety and other challenges when telling others about their diagnosis - Concealment is something individuals with illnesses may constantly work on to appear normal - Selective//partial disclosure is related to wanting support, ensuring safety, and maintaining well-being - Individuals reported that individuals responded more positively to disclosure completed in an educated manner
To Say or Not to Say: A Qualitative Study on the Disclosure of Their Condition by Human Immunodeficiency Virus–Positive Adolescents Michaud, Suris, Kahlert *et al*. (2009)^24^	Human Immunodeficiency Virus–Positive (HIV)	To investigate why and the extent to which adolescents choose to disclose or not disclose their illness	Switzerland	29 participants between the ages of 12 and 20 years old 7 = male 22 = female 20 participants were of Swiss nationality	A qualitative appraoch involving interviews	- A fear of rejection, fear of discrimination, and desire to be normal played a factor in whether individuals chose to disclose their diagnosis - A few participants recognized that concealing their illness contributed to the stigmatization of the illness and can make their life more difficult
Diabetes disclosure strategies in adolescents and young adult with type 1 diabetes Pihlaskaria, Andersona, Eshtehardi, *et al*. (2020)^25^	Type 1 Diabetes	To contribute to clinical interventions and social support by examining how adolescents and young adults disclose their illness to others	United States	16 participants aged 12 to 25 10 = male 6 = female 7 participants were white 4 participants were African- American 5 participants were Latino	A qualitative study which utilized semi-structured interviews	- Passive disclosure is a form of disclosure where others disclose the illness of individuals to others or disclosure was provided through observations of self-management task - Participants described taking a straightforward approach when engaging in open disclosure - Open disclosure was associated with reasons such as support from others, educating peers, and assistance in an event of an emergency
The centrality of disclosure decisions to the illness experience for youth with chronic conditions: A qualitative study Woodgate, Tennent, Barriage, *et al*. (2020)^4^	Chronic Illnesses: arthritis, asthma, benign brain tumours, Crohn’s/Colitis, cystic fibrosis, diabetes, heart conditions, kidney condition, and liver conditions	To highlight the disclosure experiences of youth who have chronic illnesses	Canada	54 participants ranging in the ages of 9 and 24 24 = male 30 = female	A qualitative approach involving open-ended interviews	- Concealment typically involved hiding feelings, making excuses, foregoing accommodations, withdrawing from social activities, limiting or avoiding markers of illness, and avoiding conversations and questions regarding illness - Status of illness and treatments influences whether an individual disclosed their illness - Some participants engaged in non-verbal or passive disclosure - Participants also described parents disclosing their illness for reasons related to safety and well-being - A few participants reported using strategies like making a script for when they engaged in disclosure - Disclosure was more likely to be avoided if the process seemed to be complex, such as in events where proof of illness is needed

### Study design

Various research designs and methods were used to investigate disclosure among children, adolescents, and young adults. Qualitative studies were the most popular method (n = 7). These methods include semi-structured and structured interviews. Mapping of peer relationships, photographs, and a focus group were also used. Three out of the eleven articles (n = 3) found were reviews of literature. One article (n = 1) used a mixed-method approach to collect data.

### Demographics

The inclusion criteria stated that articles must have participants under the age of 25 who have a chronic illness. A few exceptions were made. For example, although two literature reviews stated that they are examining children, adolescents and/or young adults, the age ranges were not specified. Due to the limited literature, the way these articles identified the audience in the studies that were discussed, and the richness of information that reviews can provide, we decided to include these articles. Most studies, excluding those that conducted reviews of literature, had samples that included roughly equal numbers of female and male participants. However, only four articles mentioned the ethnic backgrounds of their participants. In these studies, most participants were from African American and Latino backgrounds.

### Multiple chronic illnesses

Various chronic illnesses were identified and investigated in the literature. The most common were inflammatory bowel disease (n = 3) and epilepsy (n = 3). Three studies focused on sickle cell disorder (n = 1), human immunodeficiency virus (HIV) (n = 1), and diabetes (n = 1). Two articles investigated chronic illnesses generally (n = 2). The illnesses investigated in these articles included arthritis, asthma, benign brain tumors, Crohn’s/colitis, cystic fibrosis, heart conditions, kidney conditions, liver conditions spina bifida, a rheumatologic disorder, and a condition requiring renal dialysis or transplant.

### Main themes and findings

**Figure 2.**
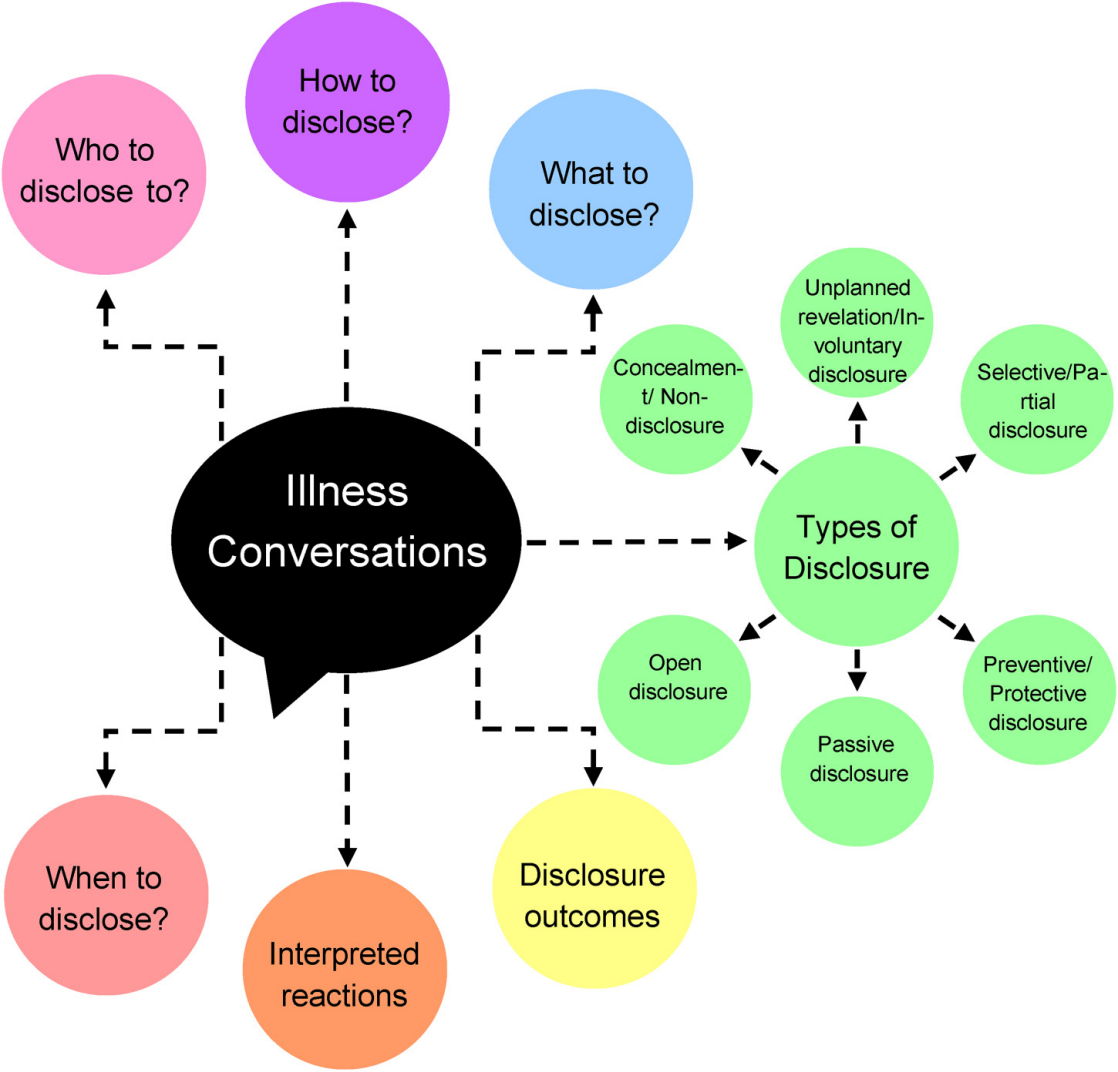
Concept chart illustrating the main themes that emerged from the review of literature.

We found that disclosure is a complex process that encompasses many factors such as the reaction of others. Many claimed this process was one of the most challenging tasks they faced.^1^ Seven themes about disclosure among peers emerged. These themes consist of: types of disclosure, who, how, what, when, reactions to disclosure, and potential outcomes (Figure 2). It should be noted that a few of these categories may overlap or be very similar to one another. The reason why there is some overlap is due to the incredibly nuanced and complex nature of illness disclosure, with factors such as time, setting, and relationships all impacting disclosure. However, it should be noted that each of the seven themes are conceptually distinct from every other theme and these differences will be discussed.

**Types of Disclosure.** All articles report different types of disclosure. Concealment or non-disclosure and open disclosure were the most commonly discussed. Some of these articles used different names for the types of disclosure or reported on disclosure more generally. One authorship team developed 5 types of disclosure.^23^ Building from these categories, 6 forms of disclosure were developed through this literature review; concealment/non-disclosure, unplanned revelation/involuntary disclosure, selective/partial disclosure, preventive/protective disclosure, passive disclosure, and open disclosure. The category of passive disclosure was added to the existing categories developed by the same research team.^23^

***Concealment/non-disclosure.*** This type of disclosure was reported in 10 articles. Although some children have a great number of friends, concealment/non-disclosure was one of the most common forms of disclosure.^6,13,22^ One research team defines concealment as children not disclosing their condition and deliberately hiding their illness from others.^23^ Children will do this by pushing their physical limitations, avoiding feelings or discussions about their illness, withdrawing from social events, creating excuses, and foregoing accommodations.^4^ Youth will also go out of their way to avoid displaying the markers of their illnesses, such as hiding medical bracelets from their peers. Children, adolescents, and young adults chose to not disclose or conceal their illness for a number of reasons, such as wanting to be ‘normal,’ avoiding negative reactions, and wanting to maintain privacy and control over the illness.^4,24,25^ Similarly, participants from other studies report fear of being rejected, socially excluded, judged, weak, teased, bullied, or treated differently.^6,10,14,20,21,23,24^ Others simply did not see the need to tell their peers because the illness did not play a role in their relationship.^6,10^ Interestingly, a few studies reported that non-disclosure was closely related to how one feels identified with their illness.^10,21,23^ Furthermore, a few studies described the process of informing others about their condition as an exhausting burden, since youth with chronic illnesses had to explain, answer questions, and correct inaccurate assumptions.^4,10,25^ As a result, children, adolescents, and young adults may avoid disclosing their illness to others. The efforts of children to conceal their illness and be seen as ‘normal’ can result in great stress, guilt, and may also worsen the condition.^4,14^

***Unplanned revelation/involuntary/forced disclosure.*** This disclosure was described in 4 articles. This form of disclosure occurs when the child, adolescent, or young adult had little control over their illness being disclosed.^4,10,14,23^ This can occur if peers witness their illness, a third party discloses their condition, or through situational cues such as frequent school absences to attend the hospital.^20,23^ The little control youth have with this disclosure can result in negative outcomes, such as confronting stigmatization or embarrassment.^14^

***Passive disclosure.*** Only two articles discussed passive disclosure. One research team described this method of disclosure as one where the individual with the chronic illness does not directly disclose their illness.^25^ Instead, disclosure of illness occurred via others or nonverbally. For example, a few participants in this study explained that their diabetes was common knowledge since it was disclosed to others by their parents when they were younger.^25^ This process typically occurs to ensure the well-being and safety of the child in environments where the parents are not the primary caregiver.^4^ Although the child’s input was involved or considered and they were actively involved in communicating the illness, youth expressed frustration when disclosure took place on their behalf.^4^

Non-verbal disclosure occurs when peers notice a change in behavior within the individual with the chronic illness or when the individual engages in illness self-management tasks, such as taking pills.^4,25^ One research team reported that this method of disclosure can be problematic as it can lead to a lack of discussion and control regarding the information that is gained by their peers.^4^ Passive disclosure differs from unplanned and involuntary disclosure because in involuntary disclosure, there is sometimes a “forced” reveal, such as parents disclosing without a child’s consent. In passive disclosure, the child is involved in the disclosure process and there is a purpose to the disclosure.

***Selective/partial disclosure.*** This type of disclosure was reported in 4 articles. Scholars described selective/partial disclosure as a process in which individuals carefully manage what information is given to others.^13,23^ This process sometimes includes withholding information.^20^ Children, adolescents, and young adults may choose this method of disclosure to control the narrative, and to avoid negative outcomes such as stigmatization and rejection.^14,20^

Similarly, ***Preventive/protective disclosure*** also involves individuals controlling what and how they disclose their illness. However, unlike selective/partial disclosure, this form of disclosure is usually informal and educational as it centers on educating peers on the physical and social aspects of the illness.^10,13^ This type of communication also leads to educating peers about what they should do in case of emergencies regarding the illness.^14^ This type of disclosure was reported in 6 articles.

***Open disclosure.*** Open disclosure was discussed in 8 articles. Open disclosure occurs when illness information is given to others with no restrictions.^23^ There are various reasons why children, adolescents, and young adults choose this form of disclosure, such as revealing one's true self, relating to others with similar experiences, advocating for oneself, and wanting peer support and understanding.^4,10,14,20,22,25^ Other reasons include securing accommodations for their illness, safety, answering questions, and warning peers about potential assistance they may need regarding their illness.^4,21,22,25^ Interestingly, only a small number of children chose to be open to everyone with their disclosure.^6,10^ This decision was typically made by those who were comfortable with illness and confident in their abilities.

**Who to disclose to? Audience considerations.** Who a child chooses to tell about their condition is dependent on various factors. Although children, adolescents, and young adults had robust peer networks, only a few friends were told about an individual’s illness.^6,10,13^ Youth chose to tell friends who they described as close, long-term, trustworthy, and comfortable.^6,10,13,22^ Participants were more likely to disclose their illness to peers who showed interest in their condition.^6^ Importantly, they also discussed that they are more likely to disclose their condition to a peer who has similar experiences or had a family member who had similar experiences.^6^ These youth explained that these peers would have a better comprehension of their experiences and are more likely to accept their condition and feelings, provide comfort, and connect with their feelings and experiences.

**How do individuals disclose their illness?** Participants in studies by two research teams report that the responsibility of disclosure shifted from their parents to them as they got older and matured.^4,6^ Before a potential disclosure, the advantages and disadvantages were considered.^6^ When choosing to disclose an illness to a peer, individuals expressed that they take into account many factors, such as the setting and time.^4^ Interestingly, individuals expressed that they prepared and practiced scripts to guide the process of disclosure.^4,10^ Professionals were seen as great support for guiding the process of disclosure, gathering resources, and gaining knowledge about the process of disclosure.^6,10^ However, there was very little information about whether these professionals were used or helpful for youth. Individuals tend to tell their peers about their illness verbally, behaviorally, and through written means.^6,10^ Verbal disclosure was typically completed in person. Behavioral disclosure refers to the individual participating in events related to the illness or completing self-management tasks. On the other hand, written disclosure was completed through online blogs or school essays.

**What do children and youth disclose?** When disclosing their illness, the amount of content and what content individuals disclose greatly varies.^10^ This is usually related to the purpose of the disclosure and who the audience of disclosure was. When disclosing an illness to peers, children, adolescents, and young adults tend to do this in an educational manner.^10^ They also tend to avoid details and lessen the severity of their illness. Since reasons for disclosure include sharing experiences, advocating for oneself, and wanting support and understanding, it can be assumed that the content of disclosure conversations may involve information about treatments, changes in appearance, and feelings regarding the illness.^4,21,22,25^

**When do youth disclose their illness to peers?** One author explained that individuals typically took months or longer to disclose their illness to their peers.^10^ Knowledge about the illness and severity of the illness plays a role in influencing when children disclose their illness. Children, adolescents, or young adults reported a desire to have sufficient information about their illness before the disclosure.^6,13^ Educating oneself about their illness early is linked to reduced fears regarding the illness, an increase in confidence, and an increase in control during the disclosure process.^6^ Participants explained the importance of educating themselves and that they delayed the disclosure of their illness because they did not want to convey and spread inaccurate information about their condition.^13^ The severity of the illness also played a role when the individual disclosed their illness to their peers as they had to decide whether they could ask for assistance in managing their illness.^6^ A few individuals explained that they told their peers when the time felt right.^10^ As mentioned earlier, disclosure could also occur when children, adolescents or young adults are asked about changes in their behavior and appearances.^10^ This typically resulted in preventive/protective disclosure.

**Interpreted reactions from others.** How individuals think others will react ­— and how others react to disclosure — plays a very influential role in whether an individual will disclose or continue to conceal their illness.^4,13^ Many are worried that they will confront negative reactions such as being judged, teased, pitied, stigmatized, and seen only as ill after disclosure.^10,13,14^ These fears were minimized in those who had more visible illnesses.^6^ For the most part, disclosure was met with positive reactions from peers. It resulted in peer support, empathy, sharing of similar experiences, and stronger relationships.^10,22^ A few peers reacted with more questions.^21,22^ Others reacted with disbelief or astonishment.^4,22^ One author explains that this reaction was more common among invisible illnesses.^6^ A few individuals reported that disclosure caused tensions and a loss of trust among peers.^10,23^ Unfortunately, some children, adolescents, and young adults were rejected or were viewed differently after illness disclosure took place.^10^

**Disclosure Outcomes.** There are positive and negative outcomes of disclosing chronic illnesses to peers. Some positive outcomes include peer support, strengthened relationships, self-advocacy, increased confidence, acceptance, accommodations, and connections with others by sharing experiences.^6,10,20^ On the other hand, negative outcomes such as distress, less social support, and being stigmatized were also associated with disclosure.^14^ Concealment or non-disclosure resulted in greater stress, worsened illness, embarrassment, and misunderstandings.^4,20^ However, a few individuals report that concealment reduced the spread of rumours.^21^

## Discussion and analysis

In our study, concealment and non-disclosure was the most common disclosure practice among children and youth with chronic illnesses even in the presence of a robust social support network of friends.^6,13,22^ This builds on existing literature because concealment was found to occur in many ways as has been reported in other studies, such as hiding identifiers of illness and withholding illness information.^4,23^ Children with chronic illnesses may choose to conceal their illnesses to avoid bullying.^26^ Further, in late childhood and adolescence, the most pressing developmental concern is acceptance by a peer group.^8^ If youth feel that disclosure will threaten acceptance in a peer group, they may also resist disclosure. The disclosure process was also perceived as an exhausting task.^4,10,25^ Researchers and clinicians should try to understand how fatigue, perceptions of difference, and peer acceptance threaten disclosure and be sensitive to these disclosure burdens. Efforts to reduce disclosure burdens should also be made. Disclosure interventions with youth may also help to promote more insight into the complexity of these decisions.

Forced, involuntary, and unplanned disclosure was also reported in our study. This is a novel finding as it categorizes disclosure practices discussed in the literature that held common factors of forced or involuntary actions. Sometimes, unplanned, forced, or involuntarily disclosure occurs when a third party, like a parent, discloses on behalf of their child without the child’s consent.^20,23^ In addition to promoting discourses of childism that devalue children’s competence to make decisions, forced disclosure might undermine youth’s agency and lead to embarrassment.^27,28^ Researchers and clinicians may consider making themselves aware that stigma and embarrassment stem from forced and involuntary disclosures. Unless it is absolutely necessary to ensure the child’s safety, efforts should be made to avoid forced and unplanned disclosures unless done in consultation with youth.

On the other hand, passive disclosure encompasses similar disclosure actions as involuntary disclosure. However, this form of disclosure is distinguished by its voluntary purpose.^4^ For example, for very young children, passive disclosure may occur via body language or knowledge that was shared a long time ago by parents before the child is able to remember.^25^ Further, in the absence of formal disclosure, peers or others may infer a non-verbal disclosure through observing a child with a chronic illness consuming pills, for example. Even though passive disclosure was not a common strategy, it is still very important for clinicians, parents, and academics to be aware of it. Given the lack of open discussion about the child’s illness and the potential for miscommunication, this kind of disclosure may be very problematic. Clinicians and researchers may consider developing resources for youth with chronic illnesses to have a more empowered role in the disclosing process. More critically, it is imperative that important stakeholders be aware of the importance, meaning, and power of non-verbal communication and body language.^29^ For non-verbal children with chronic illnesses, body language might be a very important communicative act.

Preventive and protective disclosure occurred in 6 articles, whereas selective disclosure was discussed in 4 articles. This builds on the existing literature because previous evidence found that these forms of disclosure occur when individuals carefully manage the information they give others.^4,10,13,14,20,23^ For example, protective and preventive disclosures can occur in classrooms to help peers know what to do in an emergency.^14^ On the other hand, selective disclosure may occur to gain support and combat stigmatization and rejection. More critically, one of the benefits of these types of disclosures is that it provides the ill child with a degree of control over what and how much to disclose. Indeed, a sense of mastery and control is important in the context of chronic illness.^30^ Health providers should make themselves aware that controlling the narrative may empower youth with a sense of agency. Educators should try to work with families to make these teachings and learnings as supportive as possible for youth.

Open disclosure was reported in 8 articles. This builds on the existing literature because previous studies show that open disclosure occurs with an ethos of transparency and honesty.^16^ Open disclosure may also occur in an effort to express the “true” self or to gain access to academic accommodations.^4,10,14,20–22,25^ Those in educational settings trying to facilitate academic accommodations for young people with chronic illnesses should do so with respect, dignity, and confidentiality in an empathic way.

Our review found that the audience context for disclosure has a significant impact on whether these youth disclose. This builds on the existing literature because previous studies found that youth tend to disclose to long-term friends and family who they trust and who appear to have an interest in their condition.^6,10,13,22^ Given that many young people with chronic illnesses may lack access to friendship quantity and quality with characteristics such as friendship warmth, more efforts might be made toward ensuring that these youth actually have the peer resources available to them so they can safely disclose their illness.^31^

Our review found that youth choose to disclose in person, in writing, or behaviorally, while also carefully considering the context and the timing of the disclosure.^4,6,10^ Additionally, depending on the purpose of disclosure, youth may disclose information about treatments and feelings regarding the illness. This builds on the existing literature because it provides insight as to what children and youth disclose and how they decide and prepare for disclosure. As the internet era continues to flourish and more blogs and websites are available to particular illness groups, this might potentially increase the likelihood of potential disclosures in written form online.^32^ Indeed, in our anecdotal observations, young people with chronic illnesses are choosing to disclose on social media platforms like TikTok and Instagram. Future researchers might investigate whether written disclosures are perceived to be easier versus face to face disclosures.

Our review found that children and youth also appear to disclose several months into the process of developing intimate relationships with others.^10^ Disclosure thus does not happen quickly. Youth are also more likely to disclose after they have accumulated knowledge about their own illness.^6,13^ In this regard, health providers and researchers might consider empowering youth with the educational resources they need to disclose. Health providers should also be aware that long-lasting friendships characterized by trust and reciprocity create the optimal conditions for disclosure.

Our review found that the likelihood of disclosure is strongly impacted by how youth imagine others to react.^4,13^ Previous investigations have not found this high degree of interpersonal engagement with the Other when making disclosure decisions. In this regard, our finding on the importance of interpersonal communication in the disclosure process for chronically ill children, is novel. Youth carefully consider the audience and their potential reaction before making disclosure decisions. Health providers should be cognizant of the masterful interpersonal communication skills that youth have when thinking about the disclosure process.

From our review, it is evident that disclosure has both positive and negative outcomes. This is a novel finding because this review provides an overview of outcomes for different types of disclosures.^4,6,10,14,20,21^ Health providers must consider both the positive and negative implications of disclosure before uniformly or uncritically encouraging patients to disclose. Indeed, despite the fact that honesty and transparency are heralded as virtues in many Euro-western cultures, disclosure does not always appear to be in the best interest of health.^1^ If disclosure results in more harm to a child, it may not be the ethical course of action to take.

Our methodological evaluation is also a novel component of this review. Methodologically, it is evident that most of the studies on illness and disclosure are qualitative in nature or reviews of literature. This type of evidence is critical given the need to understand the complexity of disclosure and to build the evidence base. However, future researchers might also consider conducting more quantitative studies on illness disclosure to better grasp the conditions that give rise to disclosure or non-disclosure. The use of arts-based research may also be employed to better understand the emotional and affective components of disclosure.^33,34^

The reported age range in the literature of children and youth with chronic illnesses is extremely wide, ranging from 0 to 25. This might be because of recruitment challenges in studies on pediatric chronic illness. Despite this obstacle, identification of developmental and age differences could have provided more insight into the disclosure process among children and youth. With this being said, future researchers might also consider exploring illness disclosure among more narrow age ranges, such as 11 to 13 or 14 to 16 to identify these differences. Additionally, many studies stated the gender of their participants. However, only a few studies stated that gender did not make a significant difference in results. As with age, future studies should explore the role of gender on illness disclosure.

### Recommendations for practice

Clinicians, academics, parents, educators, and other key stakeholders that work with chronically ill children should be aware that non-disclosure and concealment is a normative practice for these children and youth. Key stakeholders should avoid forced and involuntary disclosures and be aware that fear of difference and social exclusion is the main driver of non-disclosure. Critical stakeholders should reduce barriers to disclosure, encourage safe and inclusive spaces that are more likely to foster disclosure, consider disclosure interventions and supports, and ultimately support the autonomous disclosure agency of every child and youth.

### Limitations

There were several limitations both in the existing literature and in our review. Although the narrative review of literature type is an excellent forum in which to engage in a rich, narrative-like storying of the data that is so necessary to document the complexity of disclosure, it does not engage in evaluating the evidence which is more characteristic of systematic reviews of literature. As well, there were limitations in the existing literature base in that disclosure literature is generally quite limited with the exception of children with cancer. The disclosure literature should expand in the future across many illness types. Furthermore, although there were international studies included in this review, we did not consider how cultural differences impacted the disclosure process.

## Conclusion

Although there is some literature on self-disclosure, before this article, there was no peer-focused disclosure paper that captured the complexity and nuance of peer-based disclosure practices undertaken by children and youth with chronic illness. Indeed, narrative and storytelling about disclosure was made possible through the narrative review of literature type. We conducted a narrative review of literature on self-disclosure among children and youth with chronic illnesses using 3 academic databases. We found that concealment and non-disclosure was the most common form of disclosure and is born out of fear of judgment, ridicule, and bullying.^6,13,22^ However, involuntary disclosure, partial disclosure, and open disclosure were lesser practiced disclosure forms as well. The process of disclosure takes months and usually occurs in the context of close, intimate relationships.^6,10,13,22^ Youth heavily consider how they believe others will respond and thus disclosure is a highly interpersonal process. Disclosure is associated with both positive and negative health outcomes, such as acceptance and confidence, but also distress and stigmatization.^6,10,14,20^ Health providers and researchers should not uncritically support disclosure. Rather, they should aim to equip youth with the resources, skills, relationships, time, and abilities to make disclosure decisions that protect their agency, autonomy, and choices.
